# IMPROVE TECHNOLOGY AND EHEALTH LITERACY IN A RETIREMENT COMMUNITY VIA THE USE OF LLM-POWERED CHATBOT

**DOI:** 10.1093/geroni/igae098.4005

**Published:** 2024-12-31

**Authors:** Ray-Yuan Chung, Feng Chen, Yein Jeon, Oleg Zaslavsky

**Affiliations:** University of Washington, Seattle, Washington, United States; University of Washington, Seattle, Washington, United States; University of Washington, Seattle, Washington, United States; University of Washington, Seattle, Washington, United States

## Abstract

The low levels of technology and eHealth literacy of retirement community residents are a barrier to technology use and information retrieval. In response, we proposed the development of an LLM-powered chatbot to improve the technology and eHealth literacy of the residents. We adopted a human-centered design approach and aimed to complement the existing technological infrastructure of the community app by providing straightforward, conversational assistance. To enhance user accessibility, we emphasize the interface’s features including large text, white and black interface for vision support, voice interaction capabilities, and simplified navigation, catering to the varied technological proficiency levels within the community. The chatbot’s functionalities are divided into two primary goals: facilitating information navigation within the retirement community and enhancing technology and eHealth literacy among its residents. For information navigation, the chatbot allows users to easily access essential information such as dining menus and contact details. To improve technology literacy, the chatbot delivers simple, up-to-date guides and information on specific questions posed by the users, providing them with easy-to-follow instructions. To evaluate our chatbot, residents were asked to complete tasks during the trial session and complete a survey. The survey collected demographics, digital literacy scores, user satisfaction, and feasibility. Residents successfully utilized our chatbot to complete the tasks. Furthermore, they reported high levels of satisfaction and a strong likelihood of recommending it to peers. Therefore, we suggest that our chatbot may serve as a valuable tool for older adults with low technology and eHealth literacy.

